# Food passageway-related sequelae in the RefluxStop prospective multicenter trial: patient-centric outcomes of dysphagia, odynophagia, gas-bloating, and inability to belch and/or vomit at 5 years

**DOI:** 10.1007/s00464-025-11818-x

**Published:** 2025-06-20

**Authors:** László Harsányi, Zsolt Kincses, Milan Veselinović, Joerg Zehetner, Áron Altorjay

**Affiliations:** 1 Department of Surgery, Transplantation and Gastroenterology, Semmelweis University, Budapest, Hungary; 2 The Department of Surgery Kenezy Campus, Clinical Center of the University of Debrecen Teaching Hospital, Debrecen, Hungary; 3 Department of Minimally Invasive Upper Digestive Surgery, University Hospital for Digestive Surgery–First Surgical Hospital, Clinical Center of Serbia, Belgrade, Serbia; 4 Department of Visceral Surgery, Hirslanden Clinic Beau-Site, Bern, Switzerland; 5 Surgical Department, Fejér Country Szent György University Teaching Hospital, Szekesfehervar, Hungary

**Keywords:** Gastroesophageal reflux disease, Food passageway, Antireflux surgery, Dysphagia, Odynophagia, Gas-bloat syndrome, Inability to belch/vomit

## Abstract

**Introduction:**

Standard surgical management of GERD may result in troublesome postoperative food passageway-related sequelae (i.e., dysphagia, odynophagia, gas-bloat syndrome, inability to belch/vomit), significantly impacting quality of life. Five-year results after the RefluxStop procedure are presented, involving reconstruction of the anti-reflux barrier without encircling the food passageway, reducing such related sequelae.

**Methods:**

RefluxStop surgery was evaluated in a prospective, single-arm, multicenter study with 50 GERD subjects. This report focuses on food passageway-related outcomes. Other basic outcomes (e.g., 24-h pH, PPI usage) are presented in a separate report with brief clinical correlation herein.

**Results:**

Forty-four subjects completed 5-year follow-up; three participants were missing due to COVID-19 (i.e., two deaths and one bedbound with long-COVID) and three terminated early. Data from 3- and 4-year follow-up were carried forward in COVID-affected cases. Food passageway-related adverse events (AEs) between 2 weeks of surgical recovery and 5-year follow-up included: one case (2.1%) of dysphagia (and another case, mild dysphagia for 2 weeks postoperatively, viewed as normal recovery); one case (2.1%) of odynophagia; zero (0%) cases of inability to belch/vomit; and gas-bloating none/improved in 42 cases with only two worsening. These outcomes were well-aligned with improvement in total GERD-HRQL score (i.e., median 29.5 at baseline to 3.0 at 5 years), PPI usage (2.1%), and 24-h pH monitoring (i.e., mean 1.57% acid exposure time at 5 years).

**Conclusion:**

RefluxStop surgery resulted in a favorable profile of food passageway-related outcomes throughout the 5-year study: no AE dysphagia in 97.9% of subjects; no AE odynophagia in 97.9%; whereof at 5 years: gas-bloating none/improved in 95.7%, and no inability to belch/vomit in 100%. For clinical correlation, 97.9% of subjects did not take PPIs at 5 years. These outcomes add resolution to the overall treatment effect of RefluxStop and may show potential preference in GERD patients who prioritize minimization of postoperative sequelae.

Gastroesophageal reflux disease (GERD) is characterized by retrograde flow of gastric acid in the digestive tract that results in troublesome symptoms [1]. These include heartburn and dysphagia, inter alia, which may also recur following anti-reflux surgical management [2]. Dysphagia (swallowing difficulties) and odynophagia (pain with swallowing) are particularly unwanted given the anathema suffering incurred from the patient perspective. Like dysphagia and odynophagia, surgical manipulation of the food passageway may result in other sequelae associated with conventional anti-reflux surgery such as gas-bloat syndrome and the inability to belch and/or vomit. These are conceivably less prioritized in the study of anti-reflux procedures; however, they are nevertheless tremendously important due to their significant impact on quality of life.

Persistent dysphagia after anti-reflux surgery is quite common and occurs in 28.9% of cases at 5 years after the standard of care, Nissen fundoplication, a procedure that wraps the gastric fundus fully (360°) around the distal esophagus [2]. Acute dysphagia is likely a result of edema and inflammation but persistent symptoms may occur due to stenosis related to fundoplication [3]. Postoperative dysphagia is also common with alternative treatment options like magnetic sphincter augmentation (MSA), which results in dysphagia in up to 68% of patients at 1 year [4] and requires further healthcare utilization via esophageal dilatation for symptom relief or device removal in 5.6% and 2.7% of cases, respectively [5]. Furthermore, comparative study has shown no statistically significant benefit with MSA over Nissen fundoplication in terms of postoperative dysphagia [6], indicating that current management with conventional therapies has not appeased this prominent treatment gap. This is further accentuated in real-world settings where comorbid esophageal dysmotility is a risk factor for postoperative dysphagia with both fundoplication and MSA [3, 7, 8].

Gas-bloat syndrome is a subjectively defined sequela of anti-reflux surgery that is commonly reported [9] and characterized by bloating, abdominal pain, early satiety, nausea, and the inability to belch/vomit (i.e., a functional outcome presupposing gas-bloating) [10]. The prevalence of GERD-related abdominal bloating before surgery remains unclear, however, up to 50% has been reported [11]. After standard-of-care surgery with Nissen fundoplication, gas-bloating occurs in 52.7% of patients by 5 years [2]. Abdominal bloating after operative intervention is thought to be due to diminished gas venting from surgical manipulation of the lower esophageal sphincter (LES), but understanding of the exact mechanism is incomplete [9]. Thus, our thesis outlines that minimizing direct manipulation of the LES is the best approximation methodology for reducing abdominal bloating after surgery. Even though newer technology like MSA has been suggested as the preferred choice over standard-of-care surgery in medically refractory patients prioritizing gastrointestinal symptoms [9], the rate of gas-bloating remains high [11] and implies that LES augmentation alone (with preserved gas venting) is not a sufficient prevention methodology for this physiologically complex sequela associated with the belching process.

Loss of the ability to belch and/or vomit after anti-reflux surgery is common and appears to be closely related to gas-bloat syndrome [12], as previously stated. For instance, the ability to belch/vomit is lost in 39.8% of patients managed by Nissen fundoplication at 5 years [2]. There is no convincing data showing that physiologic function of the belching/emetic process returns with time [13] and may thus be consequential to the patient experience. Postsurgical loss of the ability to belch/vomit is thought to result from overcorrection of LES function and diminished capacity for relaxation in response to appropriate stimulation by gastric distension [14, 15].

Nissen fundoplication and MSA prioritize augmentation of the LES as a mechanism in reflux management [16, 17]. These procedures are associated with a substantial burden of postoperative sequelae like dysphagia, odynophagia, gas-bloating, and the inability to belch/vomit. Such adverse events (AEs) impose a detrimental effect on quality of life and satisfaction after surgery, resulting in a treatment gap that has arguably been overlooked. Applying improved understanding of the anatomical and physiological basis of normal processes, as well as GERD pathogenesis, is likely to optimize treatment success and employing techniques with broader attention to the anti-reflux barrier, like RefluxStop surgery, may alter the paradigm of management. Complications like dysphagia and gas-bloating are highly relevant due to their great effect on patient quality of life. For instance, dissatisfaction following laparoscopic fundoplication is most often due to new-onset symptoms such as dysphagia and gas-bloating despite improvement in GERD, as reported by Humphries and colleagues [18].

RefluxStop is a pioneering medical technology that aims to rectify all three components of the anti-reflux barrier, as defined by the most recent and evolved understanding of GERD pathophysiology from two American Foregut Society white papers [19, 20], highlighting a new treatment approach without encircling the distal esophagus. The RefluxStop device is designed to treat the root cause of acid reflux and restore the esophagogastric junction to a normal anatomic and physiological state, thus allowing the body to treat itself. Five-year data from the RefluxStop CE mark trial [21] is presented in a parallel report to this one and shows favorable results for overall patient-reported quality of life, via the GERD Health-Related Quality of Life (GERD-HRQL) score, that were validated by objective evaluation (i.e., pH testing and contrast swallow x-ray), and only one subject utilized proton pump inhibitors (PPIs) at 5 years. This study has been used both for CE mark in Europe as well as Food and Drug Administration (FDA) premarket submission with 5-year data for the United States (US); it therefore possesses extensive and high-quality data collection and analysis. The purpose of this report is to complement parallelly presented long-term evidence of RefluxStop surgery with the addition of food passageway-related side effects using detailed dysphagia, odynophagia, gas-bloating, and inability to belch/vomit data from the CE mark trial at 5 years.

## Methods

### Study design and objectives

This study was a prospective, single-arm, multicenter clinical trial with registration number NCT02759094 (https://clinicaltrials.gov/study/NCT02759094). A previous publication has outlined the study design in detail [22]. Fifty subjects (*n* = 50) with chronic GERD were included in the trial and had symptoms for ≥ 6 months, as demonstrated by 24-h pH testing with pathology indicated by percentage total time with pH < 4 in the distal esophagus. The CE-mark study excluded subjects with hiatal hernia ≥ 3 cm in size. Surgery was performed from December 2016 to September 2017 at four institutions and 5-year data has been used for a premarket approval (PMA) filing to the US FDA, ensuring a high rate of follow-up as well as well-controlled and analyzed data. Earlier follow-up or selection of different data (such as results of 24-h pH monitoring, GERD-HRQL questionnaire, PPI usage, and contrast swallow x-ray) up to 5 years have been published or are in peer review [21–24], meanwhile this article focuses on food passageway-related side effects (such as dysphagia, odynophagia, gas-bloating, and the inability to belch/vomit), parameters important from the patient perspective. Hiatal hernia size was measured with contrast swallow x-ray at baseline, an investigation that was repeated at 5 years. Data is delineated both as Per Protocol (PP) and as Full Analysis Set (FAS).

**Fig. 1:**
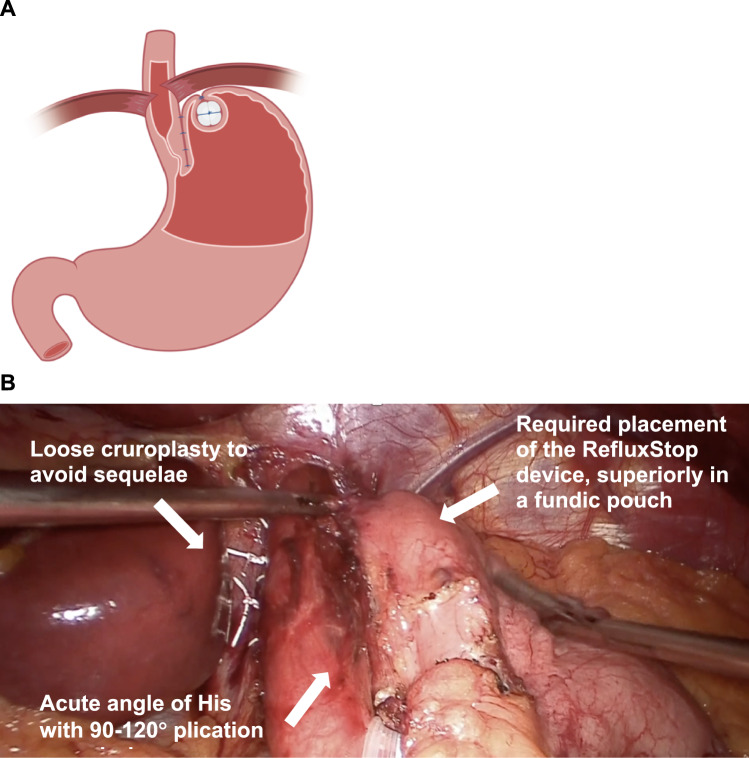
**A** Illustration showing the newly reconstructed anti-reflux barrier with an intraabdominally positioned LES, an acute angle of His and flap valve, and the RefluxStop device fully invaginated in a fundic pouch.** B** Intraoperative image showing narrow (90–120°) plication of the esophagus and stomach, placed between the vagal trunks to avoid injury, recreating the acute angle of His and gastroesophageal flap valve without encircling or compressing the distal esophagus. Narrow plication with minimal surgical manipulation of the LES is a crucial element of RefluxStop surgery that likely reduces postoperative sequelae. The device has not yet been invaginated in this figure, a necessary step to stabilize newly constructed anatomy while preventing reherniation (in applicable cases). *LES* lower esophageal sphincter

RefluxStop surgery was performed laparoscopically and involved mediastinal dissection of the esophagus with mobilization to achieve at least 4–5 cm intraabdominally, hernia reduction with repair ensuring adequate esophageal space, gastric fundus dissection (with division of four short gastric vessels), and posterior fundal dissection to attain a tension-free and floppy fundus. Esophagogastric (90–120°) plication was performed on the patient’s left side and between the vagal trunks (Fig. 1). A loose and fully closed invagination pouch was created with the RefluxStop device externally implanted on the uppermost part of the fundus.

The GERD-HRQL and Foregut symptom questionnaires were used to collect patient-reported outcomes pertaining to food passageway-related manifestations. The GERD-HRQL questionnaire evaluates symptom severity and patient satisfaction with a total score of 0–50, where higher scores indicate more severe disease. PPI use, contrast swallow x-ray, and acid exposure time (via 24-h pH testing) were investigated at 5 years to objectively determine treatment success, which is a topic of the previously mentioned and separate report [21].

### Statistical analysis

For the primary endpoint, the total GERD-HRQL score and the 95%-confidence intervals (CIs) for the percentage of patients with at least 50% improvement in total score were calculated via the Clopper-Pearson exact method formula. To test for significant differences between data of multiple follow-ups, change from baseline data were first tested for equality of variances via the Levene’s test. In case of equal variances, differences from baseline were assessed via paired t-test and, in addition, repeated measures analysis of variance (ANOVA); otherwise via the Wilcoxon signed rank test. To address multiplicity (due to testing several time points for total GERD-HRQL score) additional analyses adjusted via the Benjamini–Hochberg procedure were generated and it was confirmed that no adjusted *p*-value shifted the conclusion from statistically significant to not significant compared to unadjusted *p*-values. The significance level was 0.05.

For the secondary effectiveness endpoint, acid exposure measured by 24-h pH monitoring, the percentage of overall time with pH < 4 was analyzed by Wilcoxon signed rank test comparing baseline and follow-up visits.

## Results

Table 1 provides a summary of the key results presented in this report. At 5 years after RefluxStop surgery, patients had a favorable profile of food passageway-related side effects such as dysphagia, odynophagia, gas-bloating, and the inability to belch/vomit with prompt clinical improvement that remained stable in the long term.

**Table 1:** Summary of clinical outcomes at 5 years including three subjects’ data from 3- to 4-year follow-up that were lost to COVID-19

Food passageway-related outcomes	
Dysphagia:	97.9% (*n* = 46/47) of subjects reported no AE dysphagia after 2 weeks of surgical recovery and throughout the rest of the study. One additional subject had mild dysphagia for 2 weeks after surgery, viewed as normal surgical recovery. At 5-year follow-up, no subject had ongoing AE dysphagia
Esophageal dilatation:	Zero (0%) esophageal dilatations were performed during the entire study period (i.e., mean 5.7 years of follow-up)
Odynophagia:	97.9% (*n* = 46/47) of subjects reported no AE odynophagia during the entire study. Only one (*n* = 1/47) subject had AE odynophagia, fully recovered by 6 months
Gas-bloat syndrome:	95.7% (*n* = 45/47) of subjects had either no gas-bloating, an improvement, or equivalent symptoms at 5 years compared to baseline
Inability to belch/vomit:	100% of subjects (*n* = 41) had the ability to belch/vomit at 5-year follow-up

Data from complementary publication for clinical correlation*	
Regular daily PPI usage:*	97.9% (*n* = 46/47) of subjects did not use PPIs at 5-year follow-up, with inclusion of COVID-affected subjects (*n* = 3)
Procedure-related AEs years 1–5:*	Only two (*n* = 2) minor procedure-related AEs (mild temporary dysphagia resolved and dyspepsia) occurred between 1- and 5-year follow-up
Migration or explant:*	No migration or explant occurred during the entire study

*AE* adverse event, *PPI* proton pump inhibitor

*Detailed presentation of Effectiveness and Safety data is provided in a separate publication beyond the scope of this report [21]

### Patient population

A total of 50 subjects underwent RefluxStop surgery. Five-year follow-up was completed by 44 subjects, which were included in the PP analysis. Preoperative characteristics are as follows: mean (SD) age of 51.5 (11.8) years; mean (SD) weight of 78.2 (14.7) kg; mean (SD) body mass index (BMI) of 26.81 (4.41) kg/m^2^; male sex (56%); and mean (SD) hiatal hernia size of 2.51 (0.58) cm. AEs were followed for over 5 years.

### Subjects missing from 5-year follow-up

Forty-four (*n* = 44) of 50 subjects had data available from 5-year follow-up. Three subjects are missing due to COVID-19, two (*n* = 2) subjects died from COVID-19 and one (*n* = 1) became bed-bound with long-COVID. Results from 3- (*n* = 2) and 4-year (*n* = 1) follow-up show that all three were well-treated: 88–95% improvement in total GERD-HRQL score; cessation of PPI therapy; and no regurgitation. Food passageway-related parameters have also been included from these three subjects to reach a sample of 47/50 subjects at 5-year follow-up. Three (*n* = 3) subjects left the study in the first year (i.e., *n* = 1 at 3 months and *n* = 2 at 6 months).

### Food passageway-related outcomes

RefluxStop surgery resulted in a favorable profile of food passageway-related outcomes at 5-year follow-up, and inclusion of the three subjects lost to COVID-19 (with data carried forward from available 3- and 4-year follow-up) improved the sample size for data analysis. Five-year results of dysphagia, odynophagia, gas-bloating, and the inability to belch/vomit are presented below.

#### Dysphagia

Forty-six of 47 (97.9%) subjects did not report any AE dysphagia between 2 weeks of surgical recovery and 5-year follow-up. A detailed presentation of dysphagia incidence/severity at up to 5 years is presented in Table 2 and shows significant improvement (*p* < 0.001). One case of mild AE dysphagia occurred directly after surgery and lasted for 15 days, viewed as normal surgical recovery; and one case was reported between 1- and 5-year follow-up (i.e., both were resolved by the 5-year visit). No esophageal dilatations were performed during the entire study.

**Table 2:** Dysphagia AE—full analysis set (*n* = 50)

Dysphagia (FAS)	As per GERD-HRQL daily symptoms at baseline* (*n* = 50)		Reported AEs 0–1 year (*n* = 50)^†^		Recovered/Resolved	1-year *p*-value	Reported AEs 1–5 years (*n* = 50)^Ψ^		Recovered/Resolved	5-year *p*-value
	*n*	%	*n*	%			*n*	%		
None	39	78	49	98	–	–	49	98	–	–
Mild	6	22	1^‡^	2	Yes	< 0.001	1^¥^	2	Yes	< 0.001
Moderate	3		0		–		0		–	
Severe	2		0		–		0		–	

*AE* adverse event, *FAS* full analysis set, *GERD-HRQL* Gastroesophageal Reflux Disease Health-Related Quality of Life

*Baseline figures represent GERD-HRQL-relevant individual question score > 2 (symptoms every day)

^†^Three (*n* = 3) subjects terminated the study: two (*n* = 2) at 6 months and one (*n* = 1) at 3 months

^‡^This one (*n* = 1) subject had early-onset symptoms of mild dysphagia immediately following surgery, lasting ~ 2 weeks, seen as normal recovery

^Ψ^Three subjects lost to COVID-19 (i.e., two deaths and one bedbound with long-COVID) with use of 3- and 4-year data carried forward

^¥^This one (*n* = 1) subject had severe dysphagia before surgery with a GERD-HRQL dysphagia subscore of 5.0 which decreased to 2.0 at 3-year follow-up, indicating treatment success as opposed to the recorded AE

#### Odynophagia

Forty-six of 47 (97.9%) subjects did not report any AE odynophagia. One subject reported an AE odynophagia that lasted from surgery to ~ 6-month follow-up. A detailed presentation of odynophagia incidence/severity at up to 5 years is presented in Table 3 and shows significant improvement (*p* < 0.001). No AE odynophagia occurred after the 6-month follow-up until study completion at 5 years.

**Table 3:** Odynophagia AE—full analysis set (*n* = 50)

Dysphagia (FAS)	As per GERD-HRQL daily symptoms at baseline* (*n* = 50)		Reported AEs 0–1 year (*n* = 50)^†^		Recovered/Resolved	1-year *p*-value	Reported AEs 1–5 years (*n* = 50)^Ψ^		Recovered/Resolved	5-year *p*-value
	*n*	%	*n*	%			*n*	%		
None	39	84	49	98	–	–	49	100	–	–
Mild	3	16	1^‡^	2	Yes	< 0.001	0	0	Yes	< 0.001
Moderate	4		0		–		0		–	
Severe	1		0		–		0		–	

*AE* adverse event, *FAS* full analysis set, *GERD-HRQL* Gastroesophageal Reflux Disease Health-Related Quality of Life

*Baseline figures represent GERD-HRQL-relevant individual question score > 2 (symptoms every day)

^†^Three (*n* = 3) subjects terminated the study: two (*n* = 2) at 6 months and one (*n* = 1) at 3 months

^‡^This one (*n* = 1) subject had early-onset symptoms of moderate odynophagia immediately after surgery, lasting ~ 6 months (contrast swallow was normal)

^Ψ^Three subjects lost to COVID-19 (i.e., two deaths and one bedbound with long-COVID) with use of 3- and 4-year data carried forward

#### Gas-bloat syndrome

A significant improvement in gas-bloating symptoms was observed with the median score decreasing from 4.0 at baseline to 1.0 at 5 years and the mean score improving from 3.5 at baseline to 1.5 at 5-year follow-up. Table 4 presents the improvement of gas-bloating symptomatology scores from baseline to 5-year follow-up, based on question 9 of the GERD-HRQL questionnaire. Moreover, 95.4% (*n* = 42/44) of subjects had either no gas-bloating, an improvement, or equivalent symptoms at the 5-year visit. The three subjects lost to COVID-19 had no gas-bloating and improved (*n* = 2) or equivalent outcome (*n* = 1) at their last follow-up (3–4 years), increasing the number with favorable gas-bloating results to 95.7% (*n* = 45/47). Gas-bloating worsened in two (4.5%) subjects at 5-year follow-up.

**Table 4:** Gas-bloat syndrome (GERD-HRQL subscore)—full analysis set (*n* = 50)

Do you have bloating or gassy feeling? (*n* = 50)	Baseline	3 months	6 months	1 year	2 years	3 years	4 years	5 years	5 years + subjects lost to COVID-19* (data from years 3–4)
*n*/miss	50/0	49/1	47*/3	42/8	47/3	47/3	46/4	44/6	47^†^/3
Mean (SD)	**3.5 (1.5)**	**1.2 (1.5)**	**1.3 (1.5)**	**1.1 (1.3)**	**1.4 (1.6)**	**1.4 (1.6)**	**1.5 (1.5)**	**1.5 (1.4)**	**1.5 (1.4)**
Median	**4.0**	**1.0**	**1.0**	**1.0**	**1.0**	**1.0**	**1.0**	**1.0**	**1.0**
Min, Max	0, 5	0, 5	0, 5	0, 4	0, 5	0, 5	0, 4	0, 5	0, 5
Categorization of gas-bloating symptoms after surgery									
No symptoms at baseline and currently	–	2 (4.1%)	2 (4.3%)	3 (7.1%)	4 (8.5%)	4 (8.5%)	3 (6.5%)	2 (4.5%)	3 (6.4%)
Equivalent symptoms	–	5 (10.2%)	4 (8.5%)	2 (4.8%)	7 (14.9%)	3 (6.4%)	2 (4.3%)	3 (6.8%)	3 (6.4%)
Improved	–	39 (79.6%)	38 (80.9%)	36 (85.7%)	36 (76.6%)	38 (80.9%)	40 (87.0%)	37 (84.1%)	39 (83.0%)
Worsened	–	3 (6.1%)	3 (6.4%)	1 (2.4%)	0 (0%)	2 (4.3%)	1 (2.2%)	2 (4.5%)	2 (4.3%)
Missing	–	1	3	8	3	3	4	6	3

Bolded text highlights mean and median values

*GERD-HRQL* Gastroesophageal Reflux Disease Health-Related Quality of Life, *SD* standard deviation

Percentages are based on the number of subjects with non-missing data

*Three subjects terminated early, wherein gas-bloating improved in two subjects and was equivalent to baseline in one subject

^†^Three subjects lost to COVID-19 (i.e., two deaths and one bedbound with long-COVID) with use of 3- and 4-year data carried forward

The present analysis of gas-bloating focused on whether gas-bloating results were considered improved/favorable (i.e., did not occur, improved, was never experienced, or had equivalent symptoms to baseline) compared to worsened after surgery. The ratio of improvement/worsening was 45/2 when including the three subjects lost to COVID-19 with 3- and 4-year data carried forward.

#### Inability to belch/vomit

The question regarding the ability to belch and/or vomit was only asked at the 5-year visit in relation to follow-up pH monitoring. One hundred percent (*n* = 41) of subjects reported the retained ability to belch and/or vomit at 5 years after surgery. The preservation of the ability to belch and/or vomit at 5 years is shown in Table 5.

**Table 5:** Ability to belch/vomit—full analysis set (*n* = 50)

Are you able to belch and vomit? Question asked in relation to 24-h pH testing	Total (*n* = 50)
Year 5	
No	0 (0%)
Yes	41 (100%)
Missing	9

### Effectiveness and safety as clinical correlation

General treatment outcomes, including full safety presentation, PPI usage, GERD-HRQL score, and 24-h pH monitoring, are presented in a separate article [21] and only summarized in the clinical context of food passageway-related sequelae herein. After 5 years, 97.9% (*n* = 46/47) of subjects did not use regular daily PPI therapy, when including three subjects lost to COVID-19 with their 3–4-year results. Monitoring of 24-h pH (total time of pH < 4) was substantially decreased in all subjects with the mean acid exposure time decreasing from 16.35% at baseline to 1.57% at 5-year follow-up (*p* < 0.001), substantially within the normal threshold. The total GERD-HRQL score improved from a median score of 29.5 to 3.0 at 5-year follow-up. No explant or device migration occurred during the entire study period. Between 1- and 5-year follow-up, only two AEs occurred: mild temporary dysphagia (*n* = 1) and dyspepsia (*n* = 1).

## Discussion

This study endeavored to verify that the excellent effectiveness and safety outcomes of the RefluxStop device (with low rates of food passageway-related side effects) seen at 6 months and 1 year [22] were maintained over the long-term, in this case 5 years, to help delineate its future role in GERD management. Due to the extensive data collection in this study with analysis used both for CE-mark and FDA PMA submission of 5-year data, this evaluation possesses an extended data package, a high follow-up rate with rigorous control of data handling, and validation by third party.

When first starting to perform the RefluxStop procedure in 2016, we did not fully understand the effectiveness with which RefluxStop would be able to hinder reflux of stomach acid without affecting the food passageway. The reason for its pronounced performance may not be difficult to understand given the intrinsic logical basis of the procedure; RefluxStop in itself does not hinder acid reflux, the device and procedure instead reinstall and maintain the normal physiological and anatomical properties of the esophagogastric junction, allowing the body to treat itself naturally. After 8 years of experience with RefluxStop, we conclude that this procedure is highly effective in treating acid reflux as exhibited by the 5-year results of this study, the main data (i.e., PPI usage, GERD-HRQL score, 24-h pH monitoring, as well as contrast swallow x-ray) of which were all obtained at 5 years and presented in a separate but complementary article [21]. The focus of this report, food passageway-related side effects associated with anti-reflux procedures, shows a substantial improvement compared to the standard of care indirectly, exposition of which is provided herein. Today, the gastroenterological and surgical understanding of the mechanisms behind reflux disease (and appropriate treatment methodologies) has improved considerably [19, 20], and substantial clinical experience has been gained pertaining specifically to RefluxStop [22–33]. Not only is treatment of reflux relevant when evaluating a new procedure, it is also pertinent to treat patients with a minimum of side effects.

Standard anti-reflux surgery is troubled by adverse postoperative side effects like dysphagia, odynophagia, gas-bloating, and the inability to belch and/or vomit that are associated with encirclement and compression of the lower esophagus [34, 35], seemingly bypassed by the RefluxStop procedure. Such complications related to the food passageway are uncompromisingly relevant due to their great effect on quality of life and patient satisfaction. For instance, Humphries et al. reported that dissatisfaction after fundoplication was mostly due to new-onset symptoms such as dysphagia and gas-bloating, despite improvement in GERD [18]. With the advents of clinical experience and understanding (as well as technology), improved management of this anatomically and physiologically complex disease requires all-encompassing treatment with added/emphasized consideration of food passageway sequelae.

Food passageway-related side effects, notorious sequelae of anti-reflux surgery, occur at significant rates with the standard-of-care surgical option, Nissen fundoplication. Indirect comparison to the standard of care can be made via high-quality meta-analyses and literature reviews. Of particular relevance is a recently published and extensive literature review on Nissen fundoplication, which included 63 randomized controlled trials (RCTs) [3] and demonstrated significant long-term rates of dysphagia (28.9%), odynophagia (16%), gas-bloating (52.7%), and the inability to belch/vomit (39.8%) by 5 years [2].

Each side effect related to encircling or compression of the food passageway in this study is discussed in relation to the standard-of-care surgical option to better discern RefluxStop’s role in GERD management. Although dysphagia assumes the forefront from the clinical perspective and the surgical literature, other manifestations like odynophagia, gas-bloat syndrome, and the inability to belch/vomit exert quite a negative influence on quality of life and satisfaction with treatment as well. The manifestation of food passageway-related sequelae is probably due to the complex and dynamic nature of the esophagogastric junction, in which balance of bidirectional bolus flow is quite challenging [3], as well as the structural implications of esophageal involvement. More details are outlined below.

### Dysphagia

Acute-onset dysphagia is common after surgery and is likely due to regional edema and inflammation incurred during operation [3]. Persistent dysphagia after standard-of-care surgery occurs due to fundoplication-related stenosis (without herniation) [3]. Food passageway narrowing by this technique may result in the discomfort characteristic of dysphagia that persists in the long term, evidenced by the high incidence of postoperative dysphagia following Nissen fundoplication. According to the systematic literature review mentioned previously, postoperative dysphagia occurs in 28.9% of post-Nissen fundoplication patients after 5 years [2]. Some of the literature suggests that partial fundoplication techniques reduce the risk of dysphagia after surgery [36–39]; however, after 1 year the difference in dysphagia between total and partial fundoplication is non-significant in the mid-to-long term and more limited fundoplication may provide less favorable treatment effect for acid reflux [40, 41]. Thus, alternative techniques that have minimal impact on normal esophagogastric function are needed to optimize management while still effectively treating reflux disease.

Although MSA is a fundic-sparing intervention that does not alter gastric anatomy [3], magnetic ring augmentation nonetheless impacts the lower esophagus to the degree that 68% of patients may experience postoperative dysphagia at 1 year [4], substantially higher than the 2% rate of RefluxStop surgery in our study. Furthermore, endoscopic dilatation was performed in 5.6% of dysphagia-afflicted MSA patients in a study of 3,283 participants with a median follow-up of 1.4 years and 2.7% required MSA device removal, mostly due to dysphagia [5]. As an adjacent but relevant consideration, several articles of the MSA literature present dysphagia defined as GERD-HRQL dysphagia subscore 4 and 5 only (i.e., only very severe dysphagia is included) [42–44], to be noted when comparing to other techniques that more broadly evaluate for even milder side effects.

Furthermore, the follow-up rate in some MSA studies is, interestingly, relatively low with 75% or less completion, arguably providing results of less reliability compared to more stringently followed studies [42, 43]. Regardless, a systematic review and meta-analysis of 688 patients (i.e., *n* = 273 for Nissen fundoplication and *n* = 415 for MSA) found no statistically significant difference in postoperative dysphagia (33.9% vs. 47.1%, respectively; *p* = 0.43) as compared to fundoplication with visits up to 16 months [6]. Postoperative dysphagia after MSA is reported as lasting longer and more severe when compared to laparoscopic Nissen fundoplication, and requires dilatation for symptomatic relief [45]. This is potentially due to direct LES augmentation and the criticality of correct device-sizing (in avoidance of esophageal constriction) with MSA, also necessary for minimization of capsular fibrosis [46]. It seems, based on reported rates of food passageway-related sequelae and discussion in the literature, that the surgical implications of MSA surgery have a profound effect on food passageway function by way of possible constriction and/or regional fibrosis.

As mentioned, MSA surgery attempts to balance two opposing effects seemingly a result of its sphincter augmentation mechanism: smaller diameter sizing is associated with dysphagia risk, esophageal constriction, and erosion; and larger diameter sizing is associated with poorer treatment effect [46, 47]. Avoidance of these surgical effects may be a sound methodology for improving the patient experience and quality of life.

Dysphagia after the RefluxStop procedure is generally only caused by hiatal repair being performed too tightly. The approach by which RefluxStop is implanted, hanging freely in an invaginated pouch with limited (90–120°) plication, minimizes esophageal interaction (without encirclement or compression) and, in parallel, improves LES function via correct anatomical placement rather than augmentation, sustaining dynamic function of the esophagogastric junction (including the flap valve). This is a logical explanation for the absence of esophageal dilatation or device removal appreciated in this 5-year study. The very low rate of postoperative AE dysphagia after RefluxStop throughout the entire study (with no dysphagia at 5-year follow-up) compares favorably to 5-year rates of the surgical standard of care, Nissen fundoplication (28.9%) [2], albeit by indirect comparison. Avoidance of esophageal manipulation seems critical to improving the overall treatment effect in anti-reflux surgery even in strict settings; however, the real world encompasses difficult cases that are complicated by dysphagia-predisposing comorbidities and such patients from real-world practice are further accentuated as underserved.

The risk of dysphagia after Nissen fundoplication and MSA is particularly prominent in cases of esophageal dysmotility [7, 8, 48], posing a challenge in surgical management of GERD where conventional treatments inherently manipulate or augment the lower esophagus. For instance, functional dysphagia due to inadequate peristaltic reserve or the obstructive effect of fundoplication (that is difficult for bolus transit to overcome) occurs in the setting of ineffective esophageal motility (IEM) [3]. The MSA procedure attempts to balance the risk of severe dysphagia (with smaller diameter versions of the product) and lack of treatment effect (using larger diameter versions) [47], although the result of one outcome seems to be inversely related to the risk of the other. Given the risk of esophageal constriction with incorrect MSA device sizing and regional effects of fibrosis [46], sphincter augmentation is conceivably not optimized for minimized esophageal interaction and is not favored in those with dysmotility as per clinical practice guidance [49], due to the risk of dysphagia. Improved optimization of surgical treatment requires both reduction of dysphagia risk simultaneously with effective acid reflux control.

RefluxStop’s thesis intentionally circumvents esophageal encirclement or compression and may be a solution for these difficult-to-treat patients. Independent study from real-world practice has in fact shown favorably low rates of dysphagia in those with comorbid IEM [28, 29, 33], which encompasses short- and mid-term (up to 3 years) outcomes. The 5-year data from the present study shows that postoperative dysphagia occurs at a very low rate (2.1%) after RefluxStop surgery, a result that is sustained in the long term, which significantly improves symptoms even in severe cases.

### Odynophagia

Postoperative odynophagia as an AE occurs in 16% of cases at 5 years after Nissen fundoplication according to a literature review [2], the mechanisms of which presumably share overlap with that of postoperative swallowing difficulties. In this study, no cases of AE odynophagia occurred between 1- and 5-year follow-up (i.e., one subject reported odynophagia for 6 months after surgery which was thereafter resolved). To be noted, eight patients reported odynophagia before surgery which all resolved. This side effect is burdensome for patients and the 16% rate of painful swallowing at 5 years after the standard of care may be considered uncurbed for a satisfactory outcome. The low frequency of odynophagia after RefluxStop surgery may once again be attributed to its comprehensive and dynamic consideration of esophagogastric junction anatomy with minimization of the direct impact on the lower esophagus, thereby leaving swallowing unaffected.

### Gas-bloat syndrome

For reference, 52.7% of patients experience gas-bloating 5 years after Nissen fundoplication and almost one-third have gas-bloating quite early (at 1 year) [2]. Postoperative gas-bloat syndrome is also common 5 years after MSA and occurs in as many as 23–47% of patients [50, 51]. In this study of RefluxStop the relation of gas-bloating symptoms, expressed as a ratio of none or improved /worsened, was 42/2 subjects at 5 years, and with inclusion of the three subjects lost to COVID-19 (with 3- and 4-year data carried forward) this ratio becomes 45/2 where 95.7% (*n* = 45/47) of subjects had favorable gas-bloating results. Only two subjects (4.3%) had worsening of symptoms at 5 years compared to baseline. In comparison to more than half (52.7%) of patients with gas-bloating after standard-of-care fundoplication at the same follow-up time [2], the difference is sizeable.

Gas-bloat syndrome after anti-reflux procedures is thought to occur from impaired venting of gas through a surgically restored LES [9]. Complete understanding of gas-bloat syndrome is lacking but failure of gastric distention-induced LES relaxation, frequent swallowing of air, diminished accommodation of the stomach, and iatrogenic vagal nerve injury are proposed etiologies [9]. Those with mild cases are recommended treatment via dietary modification, avoidance of gas-inducing foods, simethicone, prokinetic agents, avoidance of aerophagia, and reassurance of spontaneous symptom resolution; however, patients are often left underserved as shown by the paucity of convincing evidence supporting the effectiveness of these strategies [10]. Regardless, the effect of gas-bloating on patient dissatisfaction is well known and a study of 1,063 patients that underwent fundoplication surprisingly found that gas-bloating was reported as the most bothersome new-onset symptom, even more so than dysphagia [18], thus meriting supplementary consideration in treatment choice for such outcomes.

Generally, patients experience alleviation of gas-bloating after anti-reflux surgery, with lower incidence observed using MSA rather than Nissen or Toupet fundoplication despite similar improvements in quality of life overall [11]. A propensity-matched analysis of Nissen fundoplication and MSA found no difference in 1- to 7-year outcomes of gas-bloating [52] and the rate of gas-bloat syndrome remains high after MSA despite its preserved belching mechanism compared to Nissen fundoplication, indicating that gas-bloating is not exclusively related to the inability to belch after fundoplication [10, 11]. Furthermore, some of the literature suggests that unfavorable results after MSA may in fact be related to gas-bloat syndrome [9] and that patients unable to achieve ≥ 50% improvement in GERD-HRQL score with complete cessation of PPI therapy are more likely to report frequent incapacitating gas-bloating symptoms [53]. The proposed mechanism of post-MSA gas-bloating is apropos the forceful distension required to open the device ring and allow for gas venting, in spite of largely preserved gastric anatomy and ability to belch/vomit with MSA [9]. A fair extrapolation is that gas-bloating is not primarily resolved by preservation of the belching process, at least one other attribute of the anti-reflux barrier must be restored for better treatment effect, and that surgical manipulation or augmentation of the LES may not be ideal. This is conceivably true given the favorable outcomes in this study in which a treatment that not only maintains normal dynamic processes of the esophagogastric junction like belching (i.e., 100% in the present study) but also addresses other deficiencies of the anti-reflux barrier without encircling or compressing the distal esophagus.

The present study indicates that RefluxStop surgery seems to be a stronger candidate in closing the treatment gap for PPI-refractory patients that are risk averse to postoperative symptoms like gas-bloating, albeit by indirect comparison the differences are pronounced. The therapeutic effect of RefluxStop surgery on all components of the anti-reflux barrier as well as its non-encircling nature may explain the low rate of gas-bloat syndrome observed. The procedure reconstitutes a dynamically functional flap valve, as part of a broader apparatus, that contributes to normal gas venting physiology in addition to restoration of LES competency, by correct intraabdominal positioning rather than augmentation.

### Inability to belch and/or vomit

RefluxStop did not induce the inability to belch and/or vomit in any patient in this study, a side effect very common in standard-of-care surgery (i.e., 39.8% at 5 years in a systematic literature review on Nissen fundoplication) [2]. Thus, the complete preservation of the belching/emetic process after RefluxStop surgery, a presupposing factor in gas-bloat syndrome and impactful function for quality of life on its own, helps minimize abdominal bloating as shown by the results discussed above.

The belching process begins with distension of the cardia that induces transient LES relaxation, allowing for air to escape into the esophagus and equalization of gastric-esophageal pressures [54]. The inability to belch/vomit after anti-reflux surgery seems to be closely related to bloating and flatulence [12] and is purportedly due to insufficient LES relaxation [54]. Considering that its manifestation after anti-reflux surgery is common, without returning over time, the utility of minimizing the structural impact on the LES should be emphasized. For instance, the level of LES augmentation that results in the inability to belch and/or vomit is similarly implicated in gas-bloating, with partial fundoplication seeming to provide more relief from such sequelae as compared to total (360°) fundoplication [55, 56]. More specifically, the mechanism behind this following fundoplication appears directly linked to LES function and its relaxation capacity after normal stimulation [14, 15]. Nissen fundoplication is thought to overcorrect mechanical deficiencies in the gastroesophageal junction at the LES, also resulting in an overly competent gastric cardia, that may incur subclinical obstruction of bolus passage at the level of fundoplication [13]. Treatment that mitigates effects on the LES and the passage of boluses may improve preservation of the belching/emetic process.

Although the range of incidence for comparison is collated from various follow-up intervals, the common belief that the inability to belch/vomit does not return with time may allow for viable comparison in the long term [13], which was necessary due to the paucity of available long-term data with MSA. As mentioned, RefluxStop surgery maintained the ability to belch/vomit in all subjects (100%) at a longer follow-up of 5 years. The preserved ability to belch and/or vomit with RefluxStop surgery appears to be completely preserved due to its non-encircling nature and restoration of the anti-reflux barrier without perceivable impact on the capacity of the LES to relax upon stimulation. This also accounts for RefluxStop’s favorable outcomes in terms of postoperative gas-bloat syndrome since they are related.

### Comparison to real-world practice of RefluxStop surgery

Overall, the available real-world data (i.e., currently > 1,200 cases performed in Europe) shows results that are similar to this pivotal study and consistently posit excellent outcomes with RefluxStop surgery as a treatment for GERD. In the pivotal trial, only hiatal hernia sizes of < 3 cm were operated on for study, meanwhile, severe reflux sufferers with complex features have also been reported on in real-world settings [25–33].

Surgeons initially adopted use of RefluxStop for the most severe cases, where the standard-of-care surgical option offers inconsistent treatment effect – these sufferers had previously been rejected for surgery due to severe esophageal dysmotility and/or large (≥ 3 cm) hiatal hernia, with severe esophagitis, dysphagia/swallowing difficulties, and Barrett’s esophagus as well. Such patients often experience deep and chronic suffering without eligibility for anti-reflux surgery, resulting in severe reflux disease. The initial patient selection for RefluxStop surgery in real-world practice had included severe sufferers, however, today all patient categories are offered RefluxStop at the institutions performing this procedure.

Despite a complex mix of demographics, the results of RefluxStop surgery in real-world settings are well-aligned with the pivotal trial, both in terms of safety and effectiveness, which indicate a robust treatment effect and favorable safety profile. As a reflection of this, real-world evidence pertaining to RefluxStop has shown excellent outcomes despite study data including the entirety of the learning curve of surgeons performing a new procedure. To date, 6.5 years after launch, the experience gained regarding circumvention of AEs and obtaining favorable results can now be passed to newer surgeons with the immediate benefits of shortening the learning curve and abstaining from the faults that early adopters underwent.

### Strengths and limitations

The strength of this safety and effectiveness study is that it is also used for both CE and FDA PMA submission (with 5-year data) purposes, providing a high-quality analysis with an extensive data package, which has been rigorously controlled. This prospective study is a landmark trial that reports the first long-term (≥ 5 years) clinical outcomes of a novel and pioneering anti-reflux procedure with a high follow-up rate of 92%, when excluding subjects that died from COVID-19 and presenting missing subjects’ data from previous visits at years 3 and 4. In addition, objective measures had been performed at 5 years, which include both 24-h pH monitoring and contrast swallow x-ray, in more than 90% of subjects remaining in the study, as presented in a separate article [21].

The focal point of this article is the clinical context provided to the overall patient experience after RefluxStop surgery by means of dysphagia, odynophagia, gas-bloat syndrome, and the inability to belch/vomit data, reported in detail. The main limitation of this study is the lack of a control group. Although the currently published literature from real-world clinical experience is growing rapidly, providing a basis for broader safety and effectiveness outcome comparisons, additional clarity will be provided by head-to-head study with the standards of care. Several ongoing retrospective studies inclusive of patients from real-world settings are also underway and may illustrate RefluxStop’s treatment effect in terms of food passageway-related outcomes in those with complicated disease, esophageal dysmotility, and hiatal hernia > 3 cm being particularly relevant. Nevertheless, RefluxStop surgery could be a preferred treatment option for PPI-refractory patients with GERD that prioritize minimization of dysphagia, odynophagia, gas-bloat syndrome, and inability to belch/vomit according to the results observed in this study.

## Conclusion

RefluxStop surgery exhibited a favorable profile of food passageway-related outcomes throughout the 5-year study: no AE dysphagia in 97.9% of subjects (with no esophageal dilatations performed) and no AE odynophagia in 97.9%; as well as at 5-year follow-up: no or improved gas-bloating in 95.7%; and no subjects had the inability to belch/vomit. For clinical correlation, no explant or device migration occurred during the entire study and 97.9% of subjects did not take PPI medication at 5 years. In addition, RefluxStop treats acid reflux effectively with normalization of 24-h pH values maintained at 5 years, as detailed further in a separate but complementary article.

The clinical outcomes of the RefluxStop procedure are due to restoration of the dynamic function of the esophagogastric junction, including all three elements of the anti-reflux barrier. This facilitates LES function without encircling the esophagus and the device is invaginated in a free-hanging stomach pouch, resulting in minimal surgical manipulation of the LES (i.e., a potential aggravant of food passageway-related sequelae). This logic is underscored by the consistency between excellent quality-of-life improvements, pH monitoring results, and PPI usage in relation to food passageway-related outcomes at 5 years after RefluxStop surgery.

The indirect comparisons made in the discussion show striking differences between RefluxStop and the standard of care, Nissen fundoplication, and such results should not be ignored even in an indirect, naïve mode of comparison. These long-term, sustained outcomes add resolution to the overall treatment effect of RefluxStop, potentially being a preferred treatment option in GERD patients that prioritize minimization of postoperative food passageway-related sequelae.
